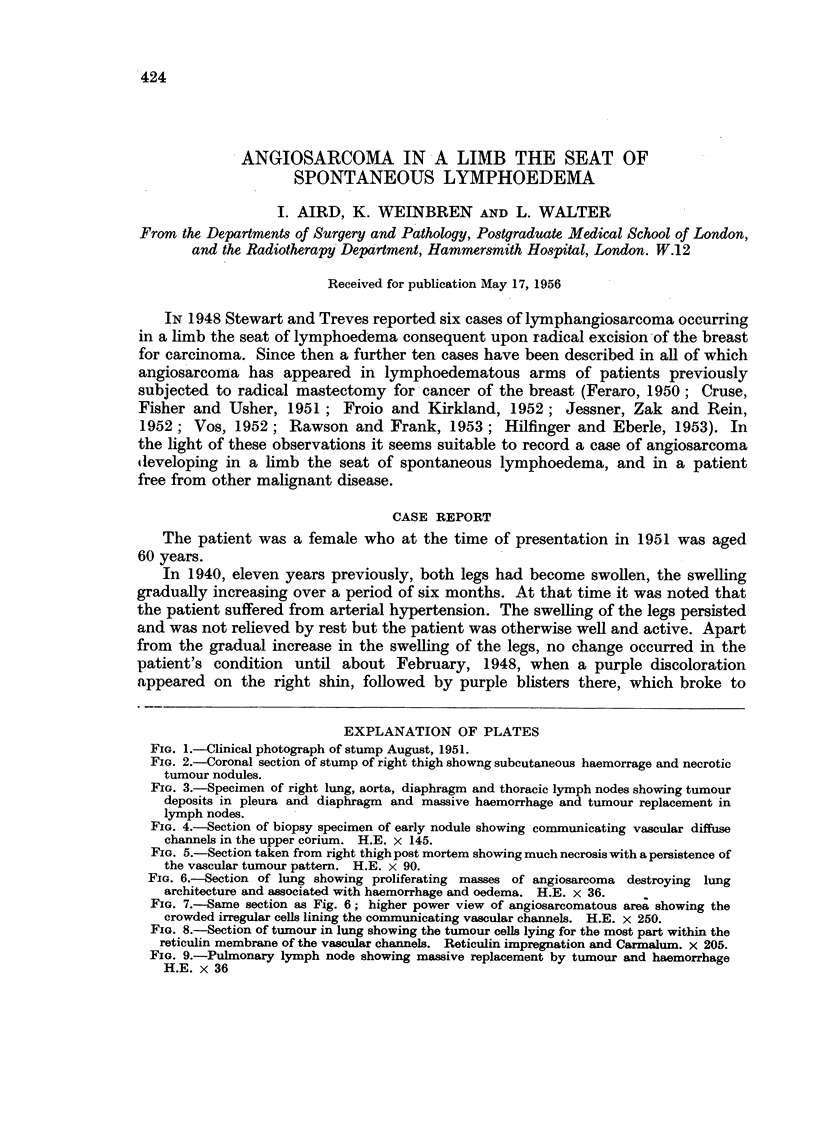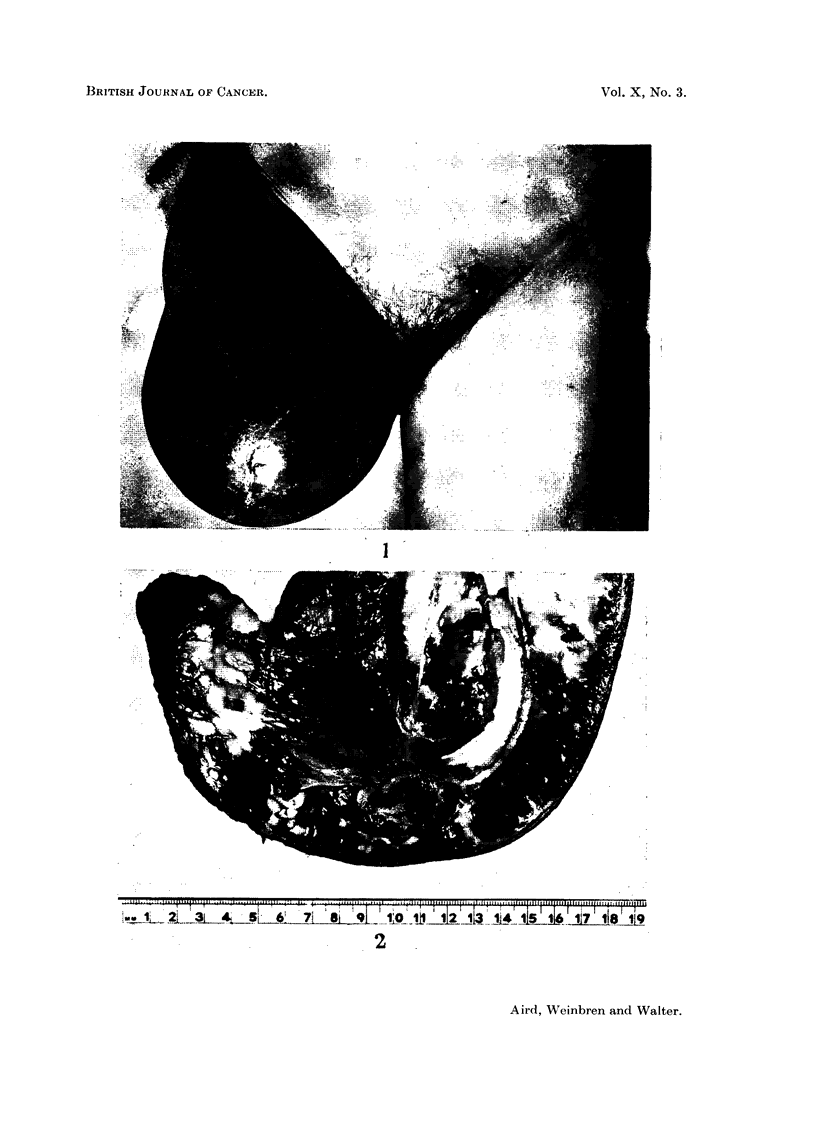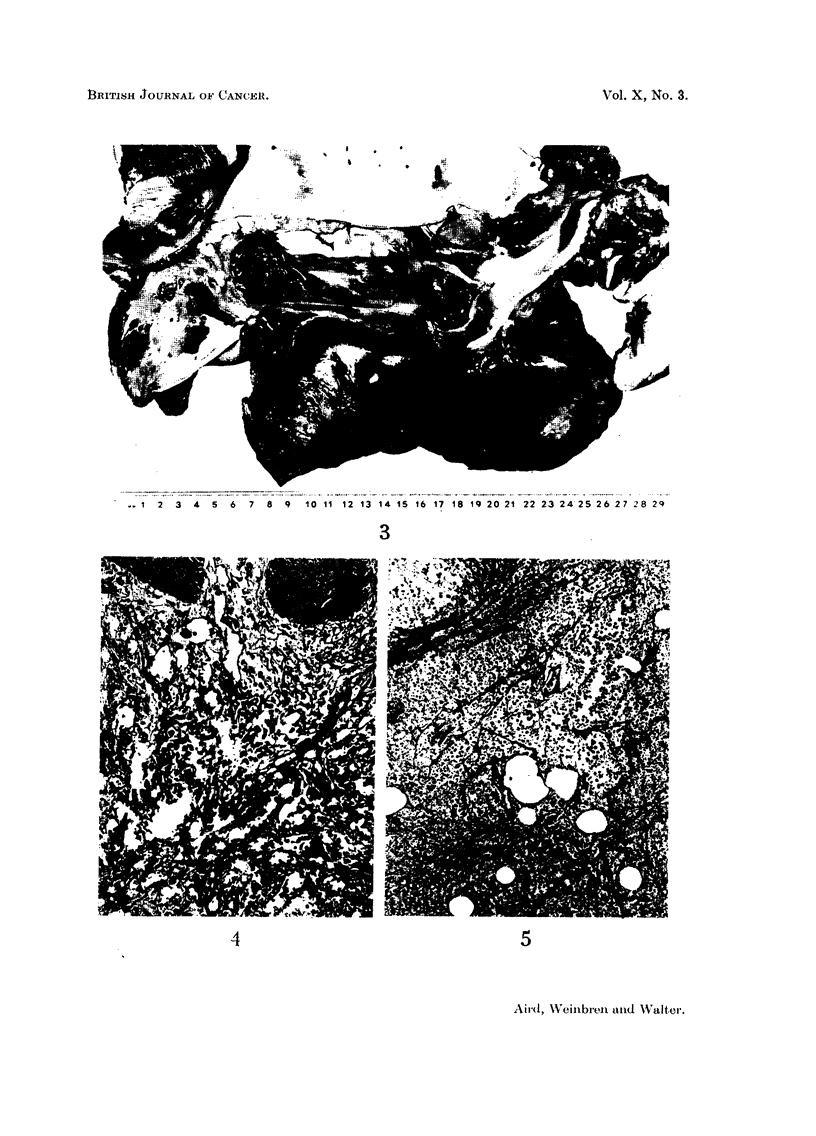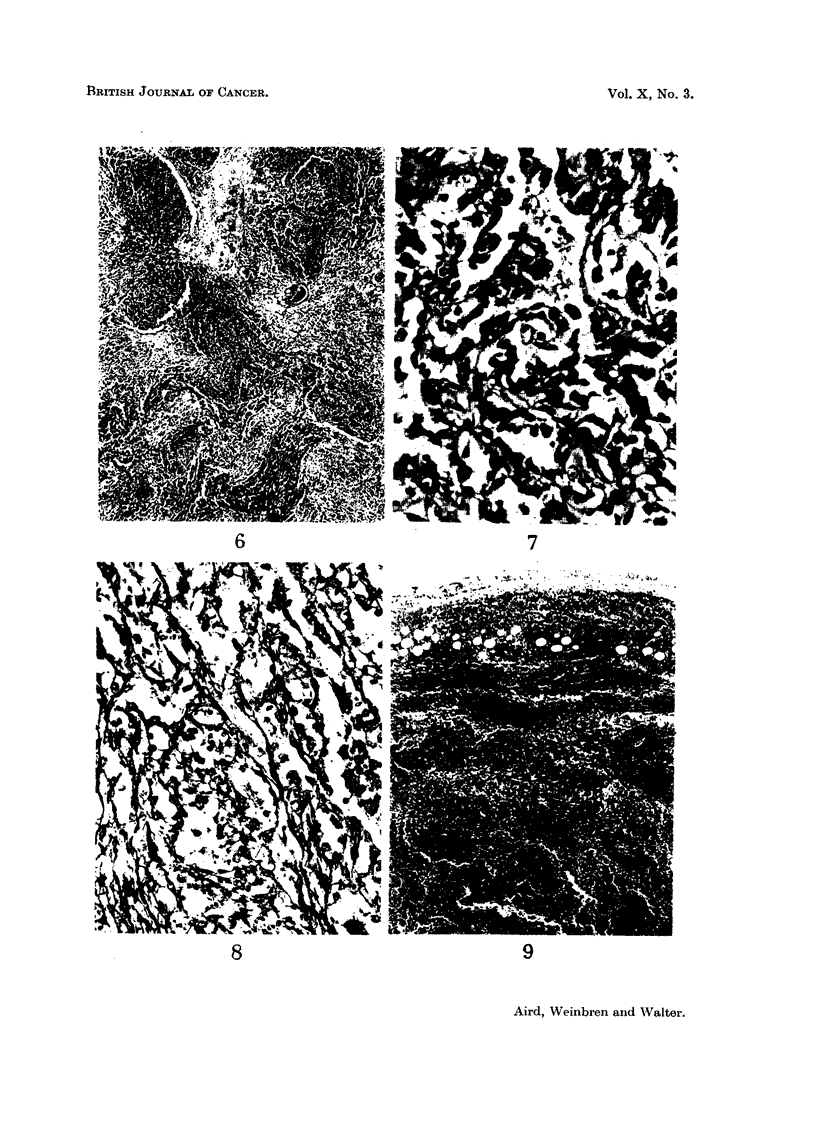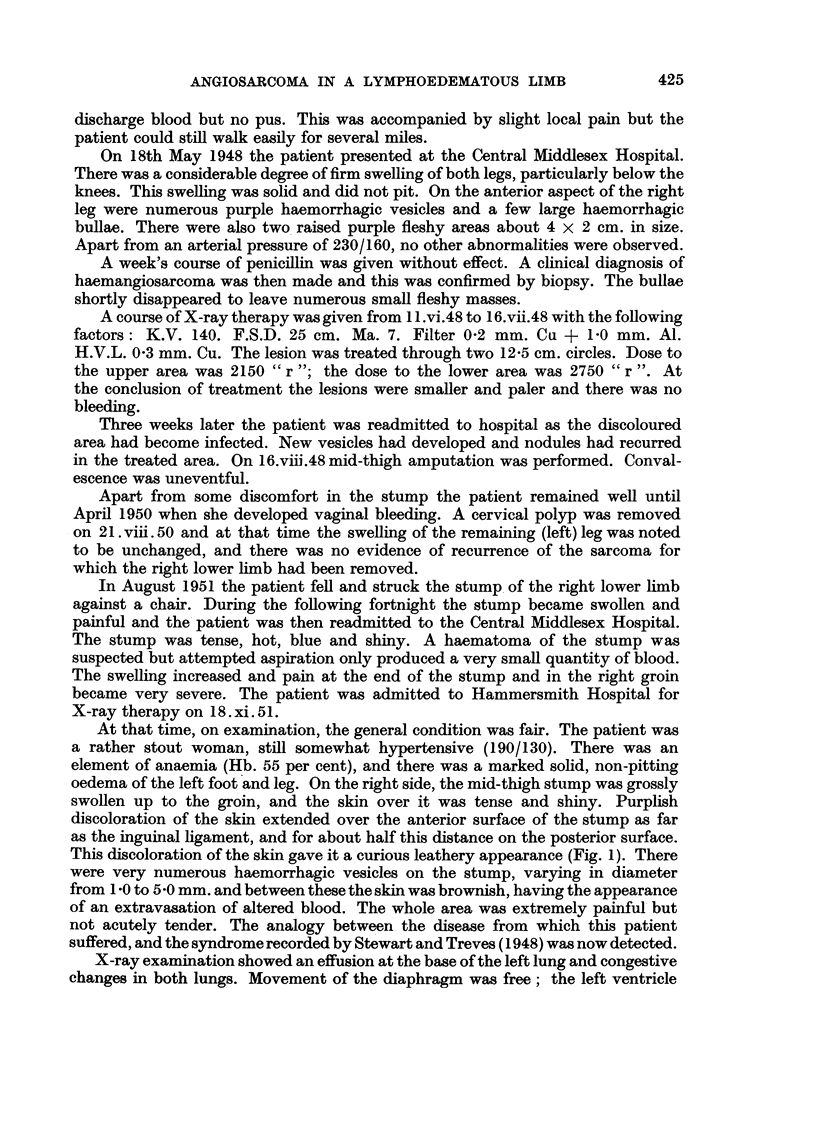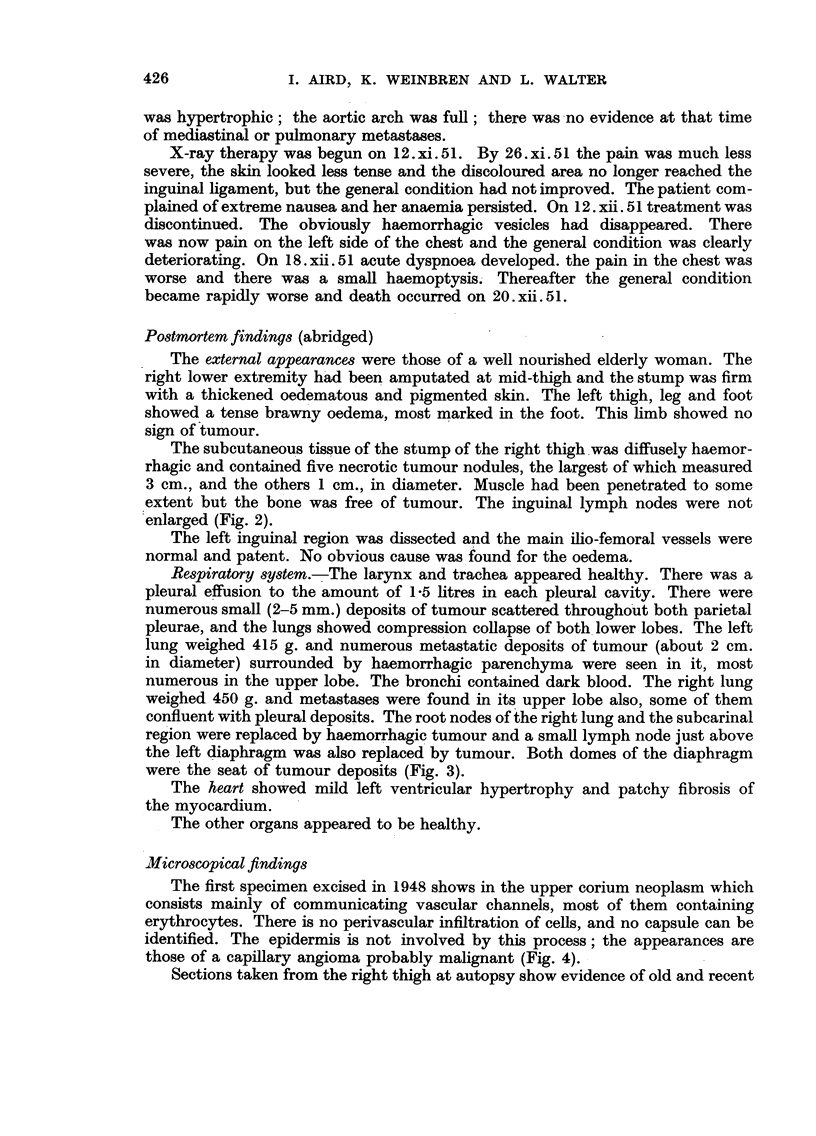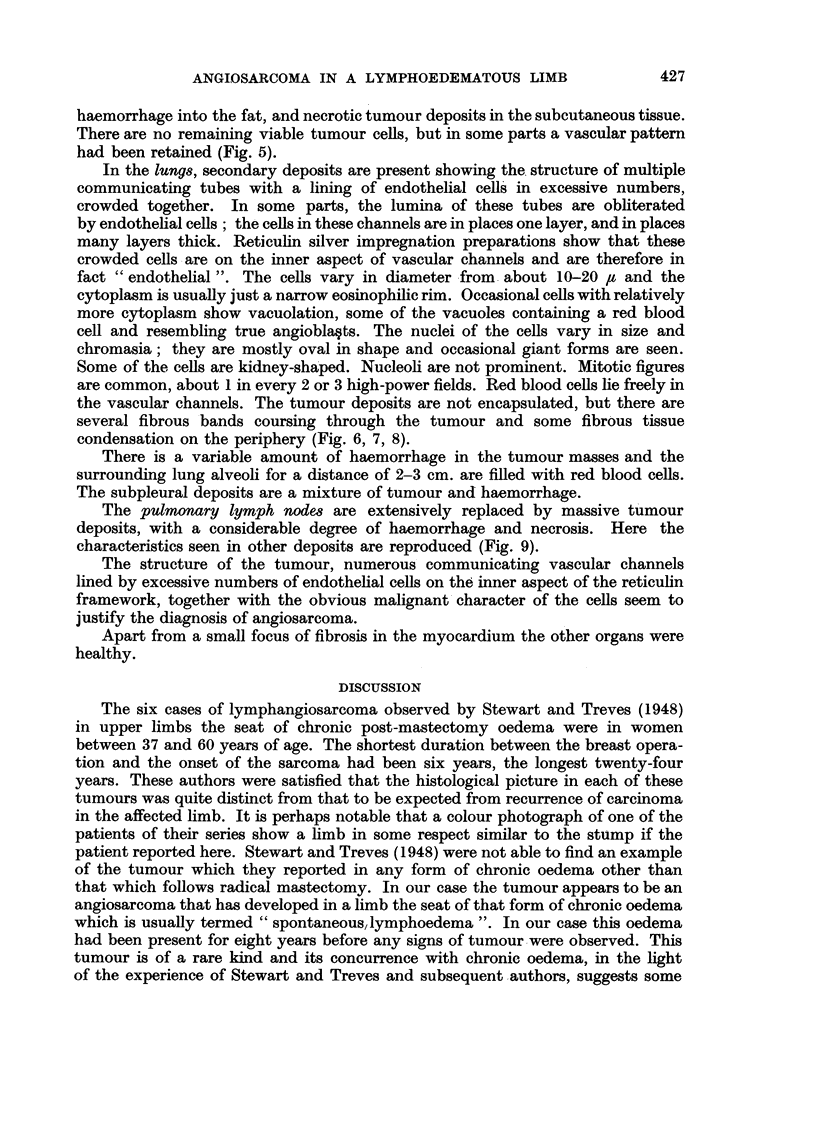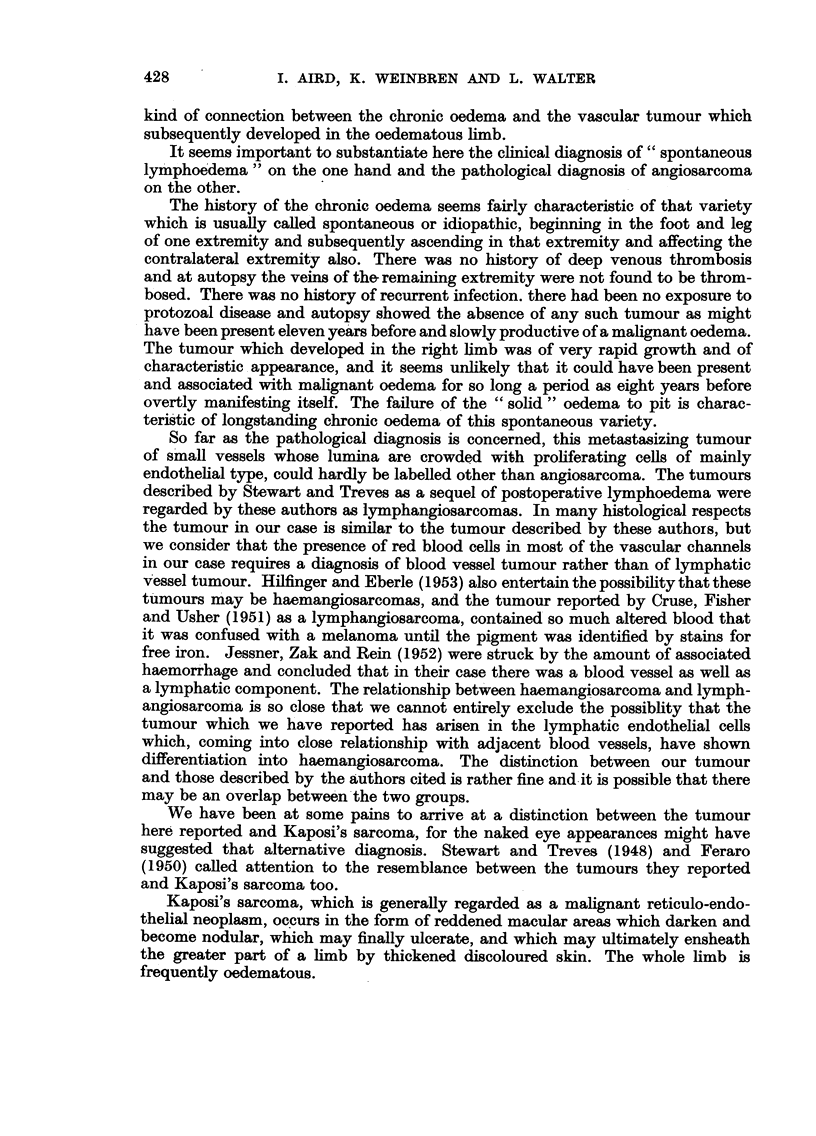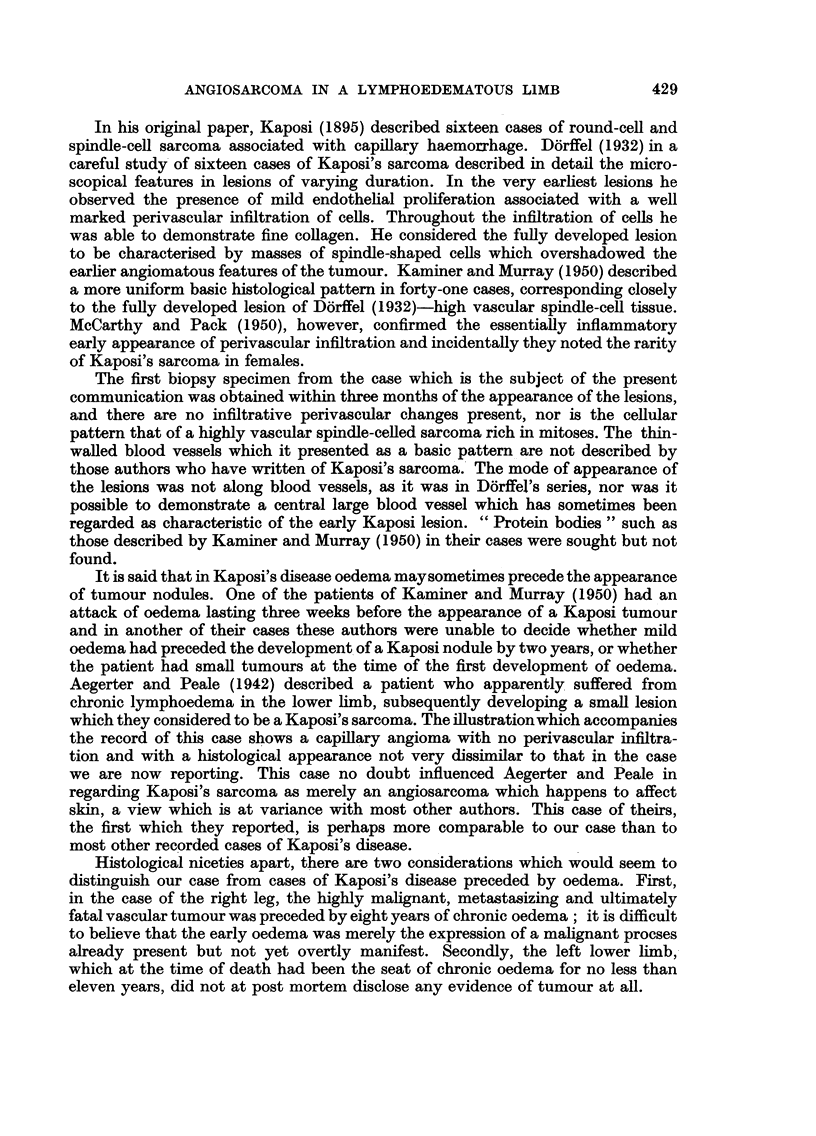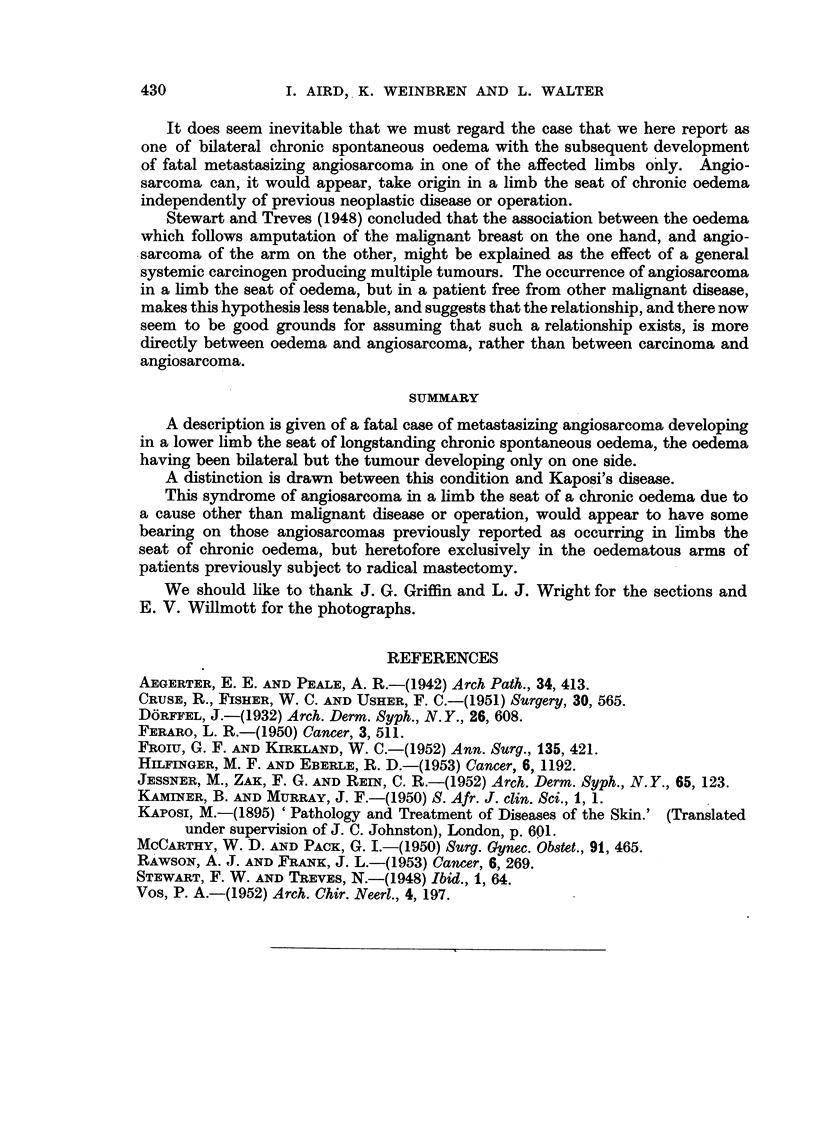# Angiosarcoma in a Limb the Seat of Spontaneous Lymphoedema

**DOI:** 10.1038/bjc.1956.49

**Published:** 1956-09

**Authors:** I. Aird, K. Weinbren, L. Walter

## Abstract

**Images:**


### ANGIOSARCOMA IN A LIMB THE SEAT OF SPONTANEOUS LYMPHOEDEMA

I. AIRD, K. WEINBREN AND L. WALTER

*From the Departments of Surgery and Pathology, Postgraduate Medical School of London, and the Radiotherapy Department, Hammersmith Hospital, London. W.12*

Received for publication May 17, 1956

In 1948 Stewart and Treves reported six cases of lymphangiosarcoma occurring in a limb the seat of lymphoedema consequent upon radical excision of the breast for carcinoma. Since then a further ten cases have been described in all of which angiosarcoma has appeared in lymphoedematous arms of patients previously subjected to radical mastectomy for cancer of the breast (Feraro, 1950 ; Cruse, Fisher and Usher, 1951 ; Froio and Kirkland, 1952 ; Jessner, Zak and Rein, 1952 ; Vos, 1952 ; Rawson and Frank, 1953 ; Hilfinger and Eberle, 1953). In the light of these observations it seems suitable to record a case of angiosarcoma developing in a limb the seat of spontaneous lymphoedema, and in a patient free from other malignant disease.

### CASE REPORT

The patient was a female who at the time of presentation in 1951 was aged 60 years.

In 1940, eleven years previously, both legs had become swollen, the swelling gradually increasing over a period of six months. At that time it was noted that the patient suffered from arterial hypertension. The swelling of the legs persisted and was not relieved by rest but the patient was otherwise well and active. Apart from the gradual increase in the swelling of the legs, no change occurred in the patient's condition until about February, 1948, when a purple discoloration appeared on the right shin, followed by purple blisters there, which broke to

---

EXPLANATION OF PLATES

FIG. 1.—Clinical photograph of stump August, 1951.

FIG. 2.—Coronal section of stump of right thigh showng subcutaneous haemorrage and necrotic tumour nodules.

FIG. 3.—Specimen of right lung, aorta, diaphragm and thoracic lymph nodes showing tumour deposits in pleura and diaphragm and massive haemorrhage and tumour replacement in lymph nodes.

FIG. 4.—Section of biopsy specimen of early nodule showing communicating vascular diffuse channels in the upper corium. H.E. × 145.

FIG. 5.—Section taken from right thigh post mortem showing much necrosis with a persistence of the vascular tumour pattern. H.E. × 90.

FIG. 6.—Section of lung showing proliferating masses of angiosarcoma destroying lung architecture and associated with haemorrhage and oedema. H.E. × 36.

FIG. 7.—Same section as Fig. 6 ; higher power view of angiosarcomatous area showing the crowded irregular cells lining the communicating vascular channels. H.E. × 250.

FIG. 8.—Section of tumour in lung showing the tumour cells lying for the most part within the reticulin membrane of the vascular channels. Reticulin impregnation and Carmalum. × 205.

FIG. 9.—Pulmonary lymph node showing massive replacement by tumour and haemorrhage H.E. × 36

1

2

3

4

5

Aird, Weinbren and Walter.

6

7

8

9

Aird, Weinbren and Walter.

discharge blood but no pus. This was accompanied by slight local pain but the patient could still walk easily for several miles.

On 18th May 1948 the patient presented at the Central Middlesex Hospital. There was a considerable degree of firm swelling of both legs, particularly below the knees. This swelling was solid and did not pit. On the anterior aspect of the right leg were numerous purple haemorrhagic vesicles and a few large haemorrhagic bullae. There were also two raised purple fleshy areas about 4 × 2 cm. in size. Apart from an arterial pressure of 230/160, no other abnormalities were observed.

A week's course of penicillin was given without effect. A clinical diagnosis of haemangiosarcoma was then made and this was confirmed by biopsy. The bullae shortly disappeared to leave numerous small fleshy masses.

A course of X-ray therapy was given from 11.vi.48 to 16.vii.48 with the following factors: K.V. 140. F.S.D. 25 cm. Ma. 7. Filter 0·2 mm. Cu + 1·0 mm. Al. H.V.L. 0·3 mm. Cu. The lesion was treated through two 12·5 cm. circles. Dose to the upper area was 2150 "r"; the dose to the lower area was 2750 "r". At the conclusion of treatment the lesions were smaller and paler and there was no bleeding.

Three weeks later the patient was readmitted to hospital as the discoloured area had become infected. New vesicles had developed and nodules had recurred in the treated area. On 16.viii.48 mid-thigh amputation was performed. Convalescence was uneventful.

Apart from some discomfort in the stump the patient remained well until April 1950 when she developed vaginal bleeding. A cervical polyp was removed on 21.viii.50 and at that time the swelling of the remaining (left) leg was noted to be unchanged, and there was no evidence of recurrence of the sarcoma for which the right lower limb had been removed.

In August 1951 the patient fell and struck the stump of the right lower limb against a chair. During the following fortnight the stump became swollen and painful and the patient was then readmitted to the Central Middlesex Hospital. The stump was tense, hot, blue and shiny. A haematoma of the stump was suspected but attempted aspiration only produced a very small quantity of blood. The swelling increased and pain at the end of the stump and in the right groin became very severe. The patient was admitted to Hammersmith Hospital for X-ray therapy on 18.xi.51.

At that time, on examination, the general condition was fair. The patient was a rather stout woman, still somewhat hypertensive (190/130). There was an element of anaemia (Hb. 55 per cent), and there was a marked solid, non-pitting oedema of the left foot and leg. On the right side, the mid-thigh stump was grossly swollen up to the groin, and the skin over it was tense and shiny. Purplish discoloration of the skin extended over the anterior surface of the stump as far as the inguinal ligament, and for about half this distance on the posterior surface. This discoloration of the skin gave it a curious leathery appearance (Fig. 1). There were very numerous haemorrhagic vesicles on the stump, varying in diameter from 1·0 to 5·0 mm. and between these the skin was brownish, having the appearance of an extravasation of altered blood. The whole area was extremely painful but not acutely tender. The analogy between the disease from which this patient suffered, and the syndrome recorded by Stewart and Treves (1948) was now detected.

X-ray examination showed an effusion at the base of the left lung and congestive changes in both lungs. Movement of the diaphragm was free ; the left ventricle was hypertrophic ; the aortic arch was full ; there was no evidence at that time of mediastinal or pulmonary metastases.

X-ray therapy was begun on 12.xi.51. By 26.xi.51 the pain was much less severe, the skin looked less tense and the discoloured area no longer reached the inguinal ligament, but the general condition had not improved. The patient complained of extreme nausea and her anaemia persisted. On 12.xii.51 treatment was discontinued. The obviously haemorrhagic vesicles had disappeared. There was now pain on the left side of the chest and the general condition was clearly deteriorating. On 18.xii.51 acute dyspnoea developed. the pain in the chest was worse and there was a small haemoptysis. Thereafter the general condition became rapidly worse and death occurred on 20.xii.51.

*Postmortem findings* (abridged)

The *external appearances* were those of a well nourished elderly woman. The right lower extremity had been amputated at mid-thigh and the stump was firm with a thickened oedematous and pigmented skin. The left thigh, leg and foot showed a tense brawny oedema, most marked in the foot. This limb showed no sign of tumour.

The subcutaneous tissue of the stump of the right thigh was diffusely haemorrhagic and contained five necrotic tumour nodules, the largest of which measured 3 cm., and the others 1 cm., in diameter. Muscle had been penetrated to some extent but the bone was free of tumour. The inguinal lymph nodes were not enlarged (Fig. 2).

The left inguinal region was dissected and the main ilio-femoral vessels were normal and patent. No obvious cause was found for the oedema.

*Respiratory system.*—The larynx and trachea appeared healthy. There was a pleural effusion to the amount of 1·5 litres in each pleural cavity. There were numerous small (2–5 mm.) deposits of tumour scattered throughout both parietal pleurae, and the lungs showed compression collapse of both lower lobes. The left lung weighed 415 g. and numerous metastatic deposits of tumour (about 2 cm. in diameter) surrounded by haemorrhagic parenchyma were seen in it, most numerous in the upper lobe. The bronchi contained dark blood. The right lung weighed 450 g. and metastases were found in its upper lobe also, some of them confluent with pleural deposits. The root nodes of the right lung and the subcarinal region were replaced by haemorrhagic tumour and a small lymph node just above the left diaphragm was also replaced by tumour. Both domes of the diaphragm were the seat of tumour deposits (Fig. 3).

The *heart* showed mild left ventricular hypertrophy and patchy fibrosis of the myocardium.

The other organs appeared to be healthy.

*Microscopical findings*

The first specimen excised in 1948 shows in the upper corium neoplasm which consists mainly of communicating vascular channels, most of them containing erythrocytes. There is no perivascular infiltration of cells, and no capsule can be identified. The epidermis is not involved by this process ; the appearances are those of a capillary angioma probably malignant (Fig. 4).

Sections taken from the right thigh at autopsy show evidence of old and recent haemorrhage into the fat, and necrotic tumour deposits in the subcutaneous tissue. There are no remaining viable tumour cells, but in some parts a vascular pattern had been retained (Fig. 5).

In the *lungs*, secondary deposits are present showing the structure of multiple communicating tubes with a lining of endothelial cells in excessive numbers, crowded together. In some parts, the lumina of these tubes are obliterated by endothelial cells ; the cells in these channels are in places one layer, and in places many layers thick. Reticulin silver impregnation preparations show that these crowded cells are on the inner aspect of vascular channels and are therefore in fact " endothelial ". The cells vary in diameter from about 10–20 $\mu$ and the cytoplasm is usually just a narrow eosinophilic rim. Occasional cells with relatively more cytoplasm show vacuolation, some of the vacuoles containing a red blood cell and resembling true angioblasts. The nuclei of the cells vary in size and chromasia ; they are mostly oval in shape and occasional giant forms are seen. Some of the cells are kidney-shaped. Nucleoli are not prominent. Mitotic figures are common, about 1 in every 2 or 3 high-power fields. Red blood cells lie freely in the vascular channels. The tumour deposits are not encapsulated, but there are several fibrous bands coursing through the tumour and some fibrous tissue condensation on the periphery (Fig. 6, 7, 8).

There is a variable amount of haemorrhage in the tumour masses and the surrounding lung alveoli for a distance of 2–3 cm. are filled with red blood cells. The subpleural deposits are a mixture of tumour and haemorrhage.

The *pulmonary lymph nodes* are extensively replaced by massive tumour deposits, with a considerable degree of haemorrhage and necrosis. Here the characteristics seen in other deposits are reproduced (Fig. 9).

The structure of the tumour, numerous communicating vascular channels lined by excessive numbers of endothelial cells on the inner aspect of the reticulin framework, together with the obvious malignant character of the cells seem to justify the diagnosis of angiosarcoma.

Apart from a small focus of fibrosis in the myocardium the other organs were healthy.

### DISCUSSION

The six cases of lymphangiosarcoma observed by Stewart and Treves (1948) in upper limbs the seat of chronic post-mastectomy oedema were in women between 37 and 60 years of age. The shortest duration between the breast operation and the onset of the sarcoma had been six years, the longest twenty-four years. These authors were satisfied that the histological picture in each of these tumours was quite distinct from that to be expected from recurrence of carcinoma in the affected limb. It is perhaps notable that a colour photograph of one of the patients of their series show a limb in some respect similar to the stump if the patient reported here. Stewart and Treves (1948) were not able to find an example of the tumour which they reported in any form of chronic oedema other than that which follows radical mastectomy. In our case the tumour appears to be an angiosarcoma that has developed in a limb the seat of that form of chronic oedema which is usually termed " spontaneous lymphoedema ". In our case this oedema had been present for eight years before any signs of tumour were observed. This tumour is of a rare kind and its concurrence with chronic oedema, in the light of the experience of Stewart and Treves and subsequent authors, suggests some kind of connection between the chronic oedema and the vascular tumour which subsequently developed in the oedematous limb.

It seems important to substantiate here the clinical diagnosis of " spontaneous lymphoedema " on the one hand and the pathological diagnosis of angiosarcoma on the other.

The history of the chronic oedema seems fairly characteristic of that variety which is usually called spontaneous or idiopathic, beginning in the foot and leg of one extremity and subsequently ascending in that extremity and affecting the contralateral extremity also. There was no history of deep venous thrombosis and at autopsy the veins of the remaining extremity were not found to be thrombosed. There was no history of recurrent infection. there had been no exposure to protozoal disease and autopsy showed the absence of any such tumour as might have been present eleven years before and slowly productive of a malignant oedema. The tumour which developed in the right limb was of very rapid growth and of characteristic appearance, and it seems unlikely that it could have been present and associated with malignant oedema for so long a period as eight years before overtly manifesting itself. The failure of the " solid " oedema to pit is characteristic of longstanding chronic oedema of this spontaneous variety.

So far as the pathological diagnosis is concerned, this metastasizing tumour of small vessels whose lumina are crowded with proliferating cells of mainly endothelial type, could hardly be labelled other than angiosarcoma. The tumours described by Stewart and Treves as a sequel of postoperative lymphoedema were regarded by these authors as lymphangiosarcomas. In many histological respects the tumour in our case is similar to the tumour described by these authors, but we consider that the presence of red blood cells in most of the vascular channels in our case requires a diagnosis of blood vessel tumour rather than of lymphatic vessel tumour. Hilfinger and Eberle (1953) also entertain the possibility that these tumours may be haemangiosarcomas, and the tumour reported by Cruse, Fisher and Usher (1951) as a lymphangiosarcoma, contained so much altered blood that it was confused with a melanoma until the pigment was identified by stains for free iron. Jessner, Zak and Rein (1952) were struck by the amount of associated haemorrhage and concluded that in their case there was a blood vessel as well as a lymphatic component. The relationship between haemangiosarcoma and lymphangiosarcoma is so close that we cannot entirely exclude the possiblity that the tumour which we have reported has arisen in the lymphatic endothelial cells which, coming into close relationship with adjacent blood vessels, have shown differentiation into haemangiosarcoma. The distinction between our tumour and those described by the authors cited is rather fine and it is possible that there may be an overlap between the two groups.

We have been at some pains to arrive at a distinction between the tumour here reported and Kaposi's sarcoma, for the naked eye appearances might have suggested that alternative diagnosis. Stewart and Treves (1948) and Feraro (1950) called attention to the resemblance between the tumours they reported and Kaposi's sarcoma too.

Kaposi's sarcoma, which is generally regarded as a malignant reticulo-endothelial neoplasm, occurs in the form of reddened macular areas which darken and become nodular, which may finally ulcerate, and which may ultimately ensheath the greater part of a limb by thickened discoloured skin. The whole limb is frequently oedematous.

In his original paper, Kaposi (1895) described sixteen cases of round-cell and spindle-cell sarcoma associated with capillary haemorrhage. Dörffel (1932) in a careful study of sixteen cases of Kaposi's sarcoma described in detail the microscopical features in lesions of varying duration. In the very earliest lesions he observed the presence of mild endothelial proliferation associated with a well marked perivascular infiltration of cells. Throughout the infiltration of cells he was able to demonstrate fine collagen. He considered the fully developed lesion to be characterised by masses of spindle-shaped cells which overshadowed the earlier angiomatous features of the tumour. Kaminer and Murray (1950) described a more uniform basic histological pattern in forty-one cases, corresponding closely to the fully developed lesion of Dörffel (1932)—high vascular spindle-cell tissue. McCarthy and Pack (1950), however, confirmed the essentially inflammatory early appearance of perivascular infiltration and incidentally they noted the rarity of Kaposi's sarcoma in females.

The first biopsy specimen from the case which is the subject of the present communication was obtained within three months of the appearance of the lesions, and there are no infiltrative perivascular changes present, nor is the cellular pattern that of a highly vascular spindle-celled sarcoma rich in mitoses. The thin-walled blood vessels which it presented as a basic pattern are not described by those authors who have written of Kaposi's sarcoma. The mode of appearance of the lesions was not along blood vessels, as it was in Dörffel's series, nor was it possible to demonstrate a central large blood vessel which has sometimes been regarded as characteristic of the early Kaposi lesion. " Protein bodies " such as those described by Kaminer and Murray (1950) in their cases were sought but not found.

It is said that in Kaposi's disease oedema may sometimes precede the appearance of tumour nodules. One of the patients of Kaminer and Murray (1950) had an attack of oedema lasting three weeks before the appearance of a Kaposi tumour and in another of their cases these authors were unable to decide whether mild oedema had preceded the development of a Kaposi nodule by two years, or whether the patient had small tumours at the time of the first development of oedema. Aegerter and Peale (1942) described a patient who apparently suffered from chronic lymphoedema in the lower limb, subsequently developing a small lesion which they considered to be a Kaposi's sarcoma. The illustration which accompanies the record of this case shows a capillary angioma with no perivascular infiltration and with a histological appearance not very dissimilar to that in the case we are now reporting. This case no doubt influenced Aegerter and Peale in regarding Kaposi's sarcoma as merely an angiosarcoma which happens to affect skin, a view which is at variance with most other authors. This case of theirs, the first which they reported, is perhaps more comparable to our case than to most other recorded cases of Kaposi's disease.

Histological niceties apart, there are two considerations which would seem to distinguish our case from cases of Kaposi's disease preceded by oedema. First, in the case of the right leg, the highly malignant, metastasizing and ultimately fatal vascular tumour was preceded by eight years of chronic oedema ; it is difficult to believe that the early oedema was merely the expression of a malignant procses already present but not yet overtly manifest. Secondly, the left lower limb, which at the time of death had been the seat of chronic oedema for no less than eleven years, did not at post mortem disclose any evidence of tumour at all.

It does seem inevitable that we must regard the case that we here report as one of bilateral chronic spontaneous oedema with the subsequent development of fatal metastasizing angiosarcoma in one of the affected limbs only. Angiosarcoma can, it would appear, take origin in a limb the seat of chronic oedema independently of previous neoplastic disease or operation.

Stewart and Treves (1948) concluded that the association between the oedema which follows amputation of the malignant breast on the one hand, and angiosarcoma of the arm on the other, might be explained as the effect of a general systemic carcinogen producing multiple tumours. The occurrence of angiosarcoma in a limb the seat of oedema, but in a patient free from other malignant disease, makes this hypothesis less tenable, and suggests that the relationship, and there now seem to be good grounds for assuming that such a relationship exists, is more directly between oedema and angiosarcoma, rather than between carcinoma and angiosarcoma.

### SUMMARY

A description is given of a fatal case of metastasizing angiosarcoma developing in a lower limb the seat of longstanding chronic spontaneous oedema, the oedema having been bilateral but the tumour developing only on one side.

A distinction is drawn between this condition and Kaposi's disease.

This syndrome of angiosarcoma in a limb the seat of a chronic oedema due to a cause other than malignant disease or operation, would appear to have some bearing on those angiosarcomas previously reported as occurring in limbs the seat of chronic oedema, but heretofore exclusively in the oedematous arms of patients previously subject to radical mastectomy.

We should like to thank J. G. Griffin and L. J. Wright for the sections and E. V. Willmott for the photographs.

### REFERENCES

Aegerter, E. E. and Peale, A. R.—(1942) *Arch Path.*, **34**, 413.
Cruse, R., Fisher, W. C. and Usher, F. C.—(1951) *Surgery*, **30**, 565.
Dörffel, J.—(1932) *Arch. Derm. Syph., N.Y.*, **26**, 608.
Feraro, L. R.—(1950) *Cancer*, **3**, 511.
Froiu, G. F. and Kirkland, W. C.—(1952) *Ann. Surg.*, **135**, 421.
Hilfinger, M. F. and Eberle, R. D.—(1953) *Cancer*, **6**, 1192.
Jessner, M., Zak, F. G. and Rein, C. R.—(1952) *Arch. Derm. Syph., N.Y.*, **65**, 123.
Kaminer, B. and Murray, J. F.—(1950) *S. Afr. J. clin. Sci.*, **1**, 1.
Kaposi, M.—(1895) 'Pathology and Treatment of Diseases of the Skin.' (Translated under supervision of J. C. Johnston), London, p. 601.
McCarthy, W. D. and Pack, G. I.—(1950) *Surg. Gynec. Obstet.*, **91**, 465.
Rawson, A. J. and Frank, J. L.—(1953) *Cancer*, **6**, 269.
Stewart, F. W. and Treves, N.—(1948) *Ibid.*, **1**, 64.
Vos, P. A.—(1952) *Arch. Chir. Neerl.*, **4**, 197.